# Mitochondrial impairment but not peripheral inflammation predicts greater Gulf War illness severity

**DOI:** 10.1038/s41598-023-35896-w

**Published:** 2023-07-12

**Authors:** Beatrice A. Golomb, Roel Sanchez Baez, Jan M. Schilling, Mehul Dhanani, McKenzie J. Fannon, Brinton K. Berg, Bruce J. Miller, Pam R. Taub, Hemal H. Patel

**Affiliations:** 1 Department of Medicine, University of California, San Diego, 9500 Gilman Drive #0995, La Jolla, CA 92093-0995 USA; 2 Present Address: San Ysidro Health Center, San Diego, CA 92114 USA; 3 VA San Diego Healthcare System and Department of Anesthesiology, University of California, San Diego, San Diego, CA 92161 USA; 4 Present Address: Avidity Biosciences, San Diego, CA 92121 USA; 5 Division of Cardiovascular Medicine, Department of Medicine, University of California, San Diego, La Jolla, CA 92037 USA

**Keywords:** Medical research, Pathogenesis

## Abstract

Gulf War illness (GWI) is an important exemplar of environmentally-triggered chronic multisymptom illness, and a potential model for accelerated aging. Inflammation is the main hypothesized mechanism for GWI, with mitochondrial impairment also proposed. No study has directly assessed mitochondrial respiratory chain function (MRCF) on muscle biopsy in veterans with GWI (VGWI). We recruited 42 participants, half VGWI, with biopsy material successfully secured in 36. Impaired MRCF indexed by complex I and II oxidative phosphorylation with glucose as a fuel source (CI&CIIOXPHOS) related significantly or borderline significantly in the predicted direction to 17 of 20 symptoms in the combined sample. Lower CI&CIIOXPHOS significantly predicted GWI severity in the combined sample and in VGWI separately, with or without adjustment for hsCRP. Higher-hsCRP (peripheral inflammation) related strongly to lower-MRCF (particularly fatty acid oxidation (FAO) indices) in VGWI, but not in controls. Despite this, whereas greater MRCF-impairment predicted greater GWI symptoms and severity, greater inflammation did not. Surprisingly, adjusted for MRCF, higher hsCRP significantly predicted *lesser* symptom severity in VGWI selectively. Findings comport with a hypothesis in which the increased inflammation observed in GWI is driven by FAO-defect-induced mitochondrial apoptosis. In conclusion, impaired mitochondrial function—but not peripheral inflammation—predicts greater GWI symptoms and severity.

## Introduction

Environmentally-triggered chronic multisymptom illnesses (CMI) impose a mounting burden on society, and share many clinical features and biomarkers—with new environmental and pharmacological triggers continuing to be identified^1–3^. Gulf War illness (GWI) is of interest both in itself, and as an exemplar of broader CMI. It may also, perhaps, serve as a model of accelerated aging: Numerous exposures such as would normally be distributed over decades (if experienced at all) were compressed into a short time span^4–6^ with marked increase in many aging-related symptoms^7–10^ and health conditions (like metabolic syndrome features^6,8,11–14^, cardiovascular disease^13,15,16^, and heightened infection risk^15^).

Nearly 700,000 U.S. troops were deployed to the 1990–1991 Persian Gulf theater of operations, of whom approximately a third developed chronic illness attributable to deployment—i.e. GWI—with symptoms encompassing fatigue, sleep problems, muscle pain and weakness, cognitive compromise, mood disturbance, diarrhea and other gastrointestinal complaints, respiratory symptoms, and dermatological disturbance^7^, among others. Evidence implicates drug, chemical, and environmental exposures which were of unprecedented variety in that conflict.

Unique exposures included pyridostigmine bromide, a nerve agent pretreatment adjunct given as a pill to ~ 250,000 of those deployed^20^; nerve gas, to which an estimated ~ 100,000 were exposed to low levels, following the Khamisiyah munitions depot demolition^17^ and possibly following other episodes^18^; botulinum toxoid vaccine^6^; a high number of multiple vaccines^5^; and oil fire smoke, among others.

New exposures included depleted uranium, with potential heavy metal and low-level radioactivity toxicity, used in that conflict for the first time to gird tanks and missiles, leading to aerosolized and sometimes shrapnel exposure following friendly fire episodes; anthrax vaccine^19^, with limited production oversight at the time; and permethrin-impregnated uniforms, among others.

Excessive exposures encompassed pesticides including acetylcholinesterase inhibiting carbamate and organophosphate pesticides^20–22^—with pesticide overexposure acknowledged to have occurred in tens of thousands^23^; nuclear biological chemical agent protection suits (NBC suits), which were used and reused after chemical alarm episodes; heat; fine sand (the finest in the world, with particles less than 0.5 microns, small enough to penetrate into the alveoli and carry toxins and microorganisms); and burn pits, among others.

Although stress had been emphasized early on as a hypothesized cause of GWI^24,25^, the ground war lasted only 4 days^26^ and many of those deployed never saw combat. Combat stress is not an independent predictor in regressions that adjust for other exposures^26^. In contrast, chemical exposures such as acetylcholinesterase inhibitor exposures have been documented to play a role^20^. Evidence for a causal role includes dose–response information^20^, as well as gene-environment interaction support^27^.

While routine laboratory tests are typically normal in veterans with GWI (VGWI), a number of objective markers have now been shown to be abnormal in GWI. Two leading hypotheses for major mechanisms underlying GWI have emerged: inflammation and mitochondrial impairment (not necessarily mutually exclusive). Inflammation has had more adherents^28–32^ perhaps in part because of greater awareness of inflammation as a contributor to health problems. CRP, a marker of peripheral inflammation, is reported to be elevated in VGWI relative to healthy controls, a replicated finding^28,29^. (There is now a human study also supporting increased neuroinflammation^32^.) However, values are typically within normal limits. Moreover, anti-inflammatory drugs like NSAIDs (non-steroidal anti-inflammatories) have not been generally reported to relieve symptoms associated with GWI. Mitochondrial impairment, long proposed^33^, has more recently had the benefit of supporting data^9,34–39^, including objective evidence of impaired bioenergetic function^34,35^ and favorable response to treatment with coenzyme Q10 (a mitochondrial coenzyme) relative to placebo. Coenzyme Q10 significantly alleviated GWI symptoms and improved objectively measured physical function, in a double-blind randomized controlled trial^9^. Among possibilities, (1) mitochondrial impairment and inflammation could concurrently and independently relate to GWI; (2) alternatively, mitochondrial impairment could relate only through the induction of inflammation e.g., via triggering of oxidative stress-induced intrinsic pathway (aka “mitochondrial”) apoptosis^40^ with resulting inflammation^41^; or (3) mitochondrial impairment could be the principal factor driving symptoms, with inflammation (e.g., arising from mitochondrial apoptosis) as a side product with limited independent contribution.

**Figure 1:**
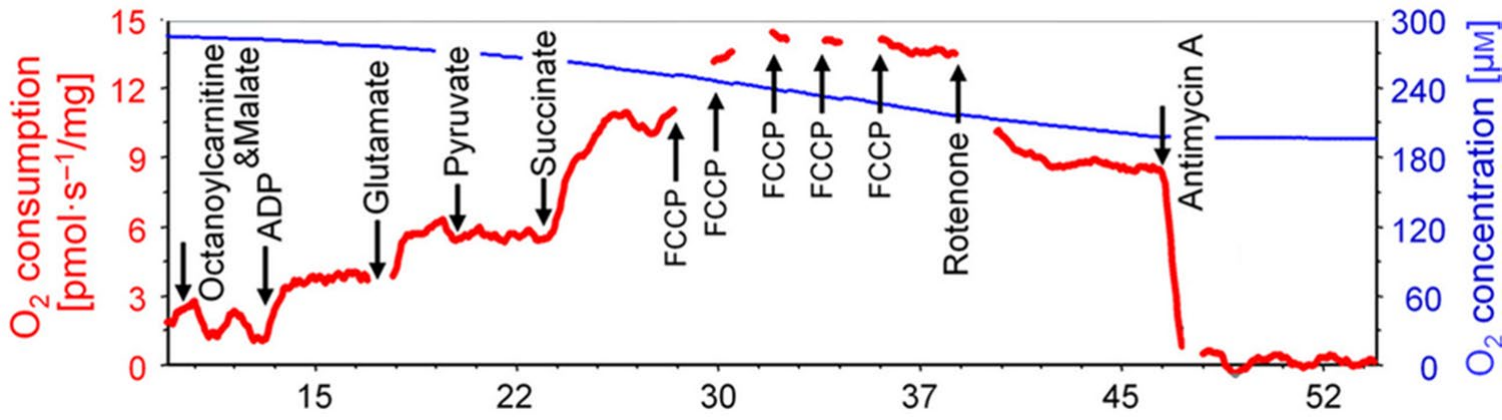
Oroboros methodology figure from the Oroboros website, illustrating addition of different substrates and inhibitors at distinct timepoints.

The present study sought to assess muscle mitochondrial indices from freshly procured muscle biopsy tissue in VGWI and healthy controls, and to assess how implicated mitochondrial indices compare to hsCRP in predicting GWI symptoms and summed symptom severity. Mitochondria are complex organelles that not only regulate cellular signaling but utilize a variety of fuel sources with coupled respiration to ATP production. Assessing coupled respiration with multiple fuel sources provides optimal assessments of mitochondrial resilience in tissues. We focused on various mitochondrial respiratory indices with electron transport complex I and II substrates with glucose and fatty acids as the fuel sources. Additional inhibitors and uncouplers were also utilized to assess residual mitochondrial respiration and maximal uncoupled respiration. The directed protocols for mitochondrial assessments systematically determine the efficiency of electron transport in muscle tissue to see how healthy and resilient the tissue is. Here we will focus more particularly on four mitochondrial indices: CI&CII&FAOCI/CIIOXPHOS refers to assessment of that aspect of mitochondrial function determined by using fatty acids as the fuel source with complex I and II substrates malate, glutamate, pyruvate, succinate and adenosine diphosphate (ADP) to drive respiration. CII&FAOETS refers to assessment of that aspect of mitochondrial function determined by using fatty acids as the fuel source with rotenone to inhibit complex I to look at complex II respiration. FAOMaxUC refers to assessment of that aspect of mitochondrial function determined by using fatty acids as the fuel source with FCCP as an uncoupler. CI&CIIOXPHOS refers to assessment of that aspect of mitochondrial function determined by using glucose as the fuel source with complex I and II substrates malate, glutamate, pyruvate, and succinate, with ADP to drive respiration. Particular attention attaches to CI&CIIOXPHOS. This study makes use of Oroboros, and to orient readers since Results are included before Methods, we provide a figure (Fig. 1), illustrating Oroboros methodology, from the Oroboros website.

## Results

Forty-two participants (21 VGWI and 21 controls) were enrolled and showed for the biopsy visit. For 1 participant (control) who showed for the biopsy visit, no biopsy was performed because hygiene issues led to concern about potential infection risk with this procedure. Of the 41 participants in whom biopsies were performed, biopsy materials were too damaged and usable data could not be secured for 2 cases and 3 controls. This provided for 36 participants (19 cases and 17 controls) with an adequate muscle tissue sample, enabling assessment of mitochondrial respiratory chain indices. Of this “full sample” of 36 participants, 28 (14 per group) contributed to a matched-pair subset analysis. In addition to the 5 for whom the designated match had a degraded sample, for 3 participants (2 cases, 1 control), their prospective match had been recruited but not yet seen at the time of the Covid-19 associated research shutdown. Matched pair analyses are subsidiary, with the focus on cross-sectional assessments of relations of objective mitochondrial and inflammatory markers to summed symptom severity, based on the full group of biopsy participants.

Nonetheless, cases and controls in the retained full sample do not significantly differ on match characteristics (Table 1). Most participants, consistent with Gulf War deployment demographics^11^, were white and male. As seen in other studies^42–44^, cases were significantly more likely to be married. Perhaps because of their challenging condition, social support is needed to add research participation to an already complicated life for those with GWI. A nonsignificant trend toward higher BMI was observed in VGWI (consistent with past reports of increased weight gain in Gulf War veterans^6^).

**Table 1:** Participant characteristics*.

	Total sample (n = 36)	Unpaired analysis			Paired analysis		
		VGWI (n = 19)	Controls (n = 17)	*P*	VGWI (n = 14)	Controls (n = 14)	*P*
Age (years)	55.9 (6.54)	56.2 (6.91)	55.6 (6.30)	0.78	55.9 (6.75)	55.4 (6.56)	0.46
% Male	91.7	89.5	94.1	0.62	92.9	92.9	1.0
% Caucasian	80.6	78.9	82.4	0.80	85.7	85.7	1.0
% Married	69.4	84.2	52.9	0.042	78.6	50.0	0.12
% Bachelor’s degree	58.3	57.9	58.8	0.96	57.1	64.3	0.70
BMI (kg/m^2^)	27.9 (3.55)	28.8 (2.39)	27.0 (4.40)	0.14	28.2 (2.01)	27.3 (4.62)	0.48
^†^Kansas # GWI domains	2.50 (2.48)	4.68 (1.11)	0.06 (0.24)	< 0.0001	4.86 (1.03)	0	< 0.0001
^†^Total Kansas GWI score	21.5 (23.4)	40.5 (16.0)	0.18 (0.53)	< 0.0001	42.1 (13.6)	0.07 (0.27)	< 0.0001
UCSD symptom score (GWI severity)	64.6 (61.9)	116.7 (36.6)	6.32 (5.65)	< 0.0001	121.5 (29.3)	5.32 (4.94)	< 0.0001

*Units are means and standard deviations for continuous variables, percentages for categorical variables. ^†^See Steele^7^.

Case–control differences in categorical variables are assessed by Chi-squared tests; for continuous variables by *t*-test (paired and unpaired, for paired and larger-sample analyses respectively).

Both hsCRP (a marker of peripheral inflammation) and highlighted mitochondrial indices showed trends toward disparities in VGWI relative to healthy controls, in the predicted direction—increased hsCRP and depressed mitochondrial function indices in VGWI (Table 2). Indices were selected as those with strong trends toward significance. All had p-values, averaging unpaired and paired analyses, of < 0.085 on one-sided testing, which is noted here to make more apparent the trends in the expected direction for these indices. (For case–control comparisons for all indices, see Supplement 1.) Continuous relations (of potential mechanistic variables to summed GWI symptom severity) are better powered than binarized comparisons and are the focus of this paper. The case–control comparisons are shown only to underscore that trends support prior reports of depressed bioenergetics and elevated inflammation in VGWI.

**Table 2:** Case–control comparisons for inflammatory and mitochondrial measures*.

	Total sample (n = 36) Mean (SD)	Unpaired analysis				Paired analysis				Description
		VGWI (n = 19) Mean (SD)	Controls (n = 17) Mean (SD)	|Dif|/SD_g_	*P*^†^	VGWI (n = 14) Mean (SD)	Controls (n = 14) Mean (SD)	|Dif|/SD_g_	*P*^†^	
HsCRP	1.65 (2.78)	2.32 (3.31)	0.90 (1.88)	0.43	0.13	2.74 (3.79)	1.02 (2.06)	0.45	0.095	Most widely used clinical marker of inflammation. The high-sensitivity version is the preferred measure for values in the < 10 mg/L range
CI&CII&FAOCI/CIIOXPHOS	56.4 (27.1)	50.7 (24.0)	62.9 (29.8)	0.51	0.23	46.0 (23.7)	72.1 (29.6)	1.10	**0.037**	Determined by using fatty acids as the fuel source with complex I and II substrates malate, glutamate, pyruvate, and succinate with ADP to drive respiration
CII&FAOETS	49.4 (18.1)	44.8 (13.7)	54.9 (21.6)	0.74	0.14	45.2 (15.5)	59.1 (22.4)	0.89	0.10	Determined by using fatty acids as the fuel source with rotenone to inhibit complex I to look at complex II respiration
FAOMaxUC	82.7 (36.1)	74.8 (29.5)	91.9 (41.7)	0.58	0.20	67.6 (26.7)	102.1 (45.5)	1.29	**0.049**	Determined by using fatty acids as the fuel source with FCCP as an uncoupler
CI&CIIOXPHOS	56.2 (22.8)	51.1 (22.8)	62.7 (21.8)	0.51	0.15	49.0 (19.8)	64.8 (24.0)	0.80	0.17	Determined by using basal MiRO5 media with complex I and II substrates malate, glutamate, pyruvate, and succinate with ADP to drive respiration

*Not every mitochondrial measure could be secured in every individual (for instance, where cytochrome c values were too high).

^†^*P*-values shown in the table are two-sided. Significant *p*-values are in bold. Prior evidence documenting peripheral inflammation (and mitochondrial impairment) in Gulf War illness might be construed as justifying one-sided testing, which better underscores that our findings are qualitatively in accord with others’ findings illustrating modest elevations in hsCRP (on average) in VGWI: one-sided testing of hsCRP differences is borderline significant and significant for unpaired and paired comparisons respectively. (For the mitochondrial indices, one-sided testing yields borderline significance for two of the indices on unpaired testing; and for paired testing, significance on two and borderline significance on the other two.)

Relations of mitochondrial indices to one another and to hsCRP are shown in Table 3. Mitochondrial fatty acid oxidation impairment strongly correlated with hsCRP in VGWI, in a fashion not observed in controls. CI&CIIOXPHOS also strongly correlated to fatty acid oxidation indices in cases, with these relations absent or markedly blunted (and nonsignificant) in controls. The most exaggerated case–control difference is in the relation between CI&CIIOXPHOS and FAOMaxUC, showing a strong and highly significant correlation in cases and essentially no correlation in controls.

**Table 3:** Correlations among hsCRP and highlighted mitochondrial indices.

	HsCRP	CI&CII&FAOCI/CIIOXPHOS	CII&FAOETS	FAOMaxUC			CI&CIIOXPHOS
**a. Total sample**							
HsCRP	1	–	–	–			–
CI&CII&FAOCI/CIIOXPHOS	− 0.44	1	–	–			–
	**0.016**						
CII&FAOETS	− 0.49	0.85	1	–			–
	**0.006**	**< 0.0001**					
FAOMaxUC	− 0.38	0.92	0.84	1			–
	**0.038**	**< 0.0001**	**< 0.0001**				
CI&CIIOXPHOS	− 0.25	0.66	0.60	0.51			1
	0.17	**0.0003**	**0.001**	**0.008**			
**b. Cases**							
HsCRP	1	–	–		–	–	
CI&CII&FAOCI/CIIOXPHOS	− 0.71	1	–		–	–	
	**0.002**						
CII&FAOETS	− 0.79	0.84	1		–	–	
	**0.0003**	**< 0.0001**					
FAOMaxUC	− 0.70	0.94	0.79		1	–	
	**0.002**	**< 0.0001**	**0.0002**				
CI&CIIOXPHOS	− 0.35	0.87	0.72		0.80	1	
	0.15	**< 0.0001**	**0.0025**		**0.0003**		
**c. Controls**							
HsCRP	1	–	–		–	–	
		–	–		–	–	
CI&CII&FAOCI/CIIOXPHOS	− 0.26	1	–		–	–	
			–		–	–	
	0.37						
CII&FAOETS	− 0.13	0.84	1		–	–	
					–	–	
	0.67	**0.0003**					
FAOMaxUC	− 0.19	0.90	0.84		1	–	
	0.52	**< 0.0001**	0.0003			–	
CI&CIIOXPHOS	0.14	0.33	0.38		0.09	1	
	0.64	0.32	0.28		0.79		

Pearson’s correlation coefficients. The coefficient is represented by the number on top and the *p*-value is represented by the number on the bottom. Significant *p*-values are in bold.

Of the candidate mitochondrial indices, CI&CIIOXPHOS was the most dissociated from hsCRP, so was selected for further comparison to hsCRP against symptoms. This dissociation aids in reducing the impact of collinearity in contrasting the mitochondrial and inflammatory indices. CI&CIIOXPHOS showed broad relationships to many symptoms and generally the strongest relation to cognitive (and certain other) symptoms. A couple of fatty acid oxidation indices related slightly more strongly to muscle indices, with CII&FAOETS the strongest: correlation coefficients (p-values) for low energy, muscle weakness and fatigue with exertion were r = − 0.41 (*p* = 0.029), r = − 0.43 (*p* = 0.021) and r = − 0.45 (*p* = 0.014) respectively. CIIMaxUC (aspect of mitochondrial function determined by using glucose as the fuel source with FCCP as an uncoupler) related to cold limbs, significantly in the total sample and with borderline significance in VGWI assessed separately: r = − 0.40 (*p* = 0.033) and r = − 0.47 (*p* = 0.065), respectively. In contrast, as Tables 4 and 5 show, cold limbs bore ~ *no* relation to CI&CIIOXPHOS.

**Table 4:** Pairwise correlations and corresponding *p*-values for CI&CIIOXPHOS and symptoms.

	Total sample (n = 32)			VGWI (n = 18)		
	r	*P*	Sign	r	*P*	Sign
Aches/pains	− 0.33	0.069*	+	− 0.32	0.20	+
Joint pain	− 0.34	0.059*	+	− 0.36	0.14	+
Muscle pain	− 0.35	0.052*	+	− 0.28	0.27	+
Headache	− 0.40	0.024*	+	− 0.41	0.091*	+
Tiredness	− 0.26	0.16	+	− 0.27	0.29	+
Sleep problems	− 0.13	0.48	+	0.05	0.84	−
Low energy	− 0.37	0.036*	+	− 0.38	0.12	+
Muscle weakness	− 0.37	0.038*	+	− 0.37	0.13	+
Post-exertion fatigue	− 0.43	0.014*	+	− 0.48	0.042*	+
Irritability	− 0.36	0.044*	+	− 0.36	0.15	+
Impatience	− 0.33	0.069*	+	− 0.29	0.25	+
Anxiety	− 0.43	0.015*	+	− 0.43	0.073*	+
Need to recheck	− 0.33	0.063*	+	− 0.24	0.33	+
Word/name recall	− 0.37	0.038*	+	− 0.38	0.12	+
Concentration problems	− 0.41	0.019*	+	− 0.46	0.058*	+
Difficulty remembering	− 0.45	0.010*	+	− 0.55	0.019*	+
Reading difficulty	− 0.39	0.029*	+	− 0.39	0.11	+
Cold limbs	− 0.03	0.86	+	0.17	0.51	−
Dry skin	− 0.44	0.011*	+	− 0.49	0.040*	+
Ringing in ears	− 0.31	0.079*	+	− 0.28	0.26	+

r = Pearson’s correlation coefficient. CI&CIIOXPHOS was secured for 32 of the 36 usable biopsies.

*P*-values with an asterisk are at least borderline significant, while bolded values have two-sided significance.

The sign is designated as + for relations in the predicted direction and − for relations in the opposite direction.

**Table 5:** Pairwise correlations and corresponding *p*-values for hsCRP and symptoms.

	Total sample (n = 36)			VGWI (n = 19)		
	r	*P*	Sign	r	*P*	Sign
Aches/pains	0.15	0.37	+	− 0.06	0.81	−
Joint pain	0.10	0.55	+	− 0.27	0.27	−
Muscle pain	0.21	0.23	+	− 0.07	0.78	−
Headache	0.05	0.78	+	− 0.16	0.50	−
Tiredness	0.34	0.044*	+	0.08	0.75	+
Sleep problems	0.20	0.25	+	− 0.16	0.50	−
Low energy	0.18	0.29	+	− 0.08	0.75	−
Muscle weakness	0.16	0.37	+	− 0.14	0.57	−
Post-exertion fatigue	0.18	0.30	+	− 0.08	0.73	−
Irritability	0.23	0.18	+	0.03	0.89	+
Impatience	0.15	0.37	+	− 0.01	0.96	−
Anxiety	0.27	0.11	+	0.17	0.48	+
Need to recheck	0.004	0.98	+	− 0.51	0.025*	**−**
Word/name recall	0.16	0.35	+	− 0.11	0.66	−
Concentration problems	0.23	0.18	+	0.039	0.87	+
Difficulty remembering	0.23	0.18	+	0.04	0.88	+
Reading difficulty	0.26	0.13	+	0.14	0.57	+
Cold limbs	− 0.10	0.57	−	− 0.35	0.15	−
Dry skin	0.20	0.25	+	0.05	0.83	+
Ringing in ears	0.12	0.49	+	− 0.10	0.68	−

r = Pearson’s correlation coefficient. *P*-values with an asterisk are at least borderline significant, while bolded values have two-sided significance. The sign is designated as + for relations in the predicted direction and − for relations in the opposite direction. Note that if symptoms are greater in VGWI than in controls, and hsCRP is greater in VGWI than in controls, this will create apparent correlations in the total sample even if the two variables do not relate to one another within VGWI—as seen here. Note that, in contrast, pairwise correlation coefficients between the symptoms and the mitochondrial index (Table 4) are similar for the total sample and for VGWI.

Tables 4 and 5 show the correlation of CI&CIIOXPHOS, and of hsCRP, to each of the 20 symptoms of the UCSD GWI symptom survey—in the total sample and in VGWI separately. As this table makes evident, symptoms show a far stronger relationship to the mitochondrial measure than to inflammation. In the total sample, 20 symptoms out of 20 show the predicted direction of relation to CI&CIIOXPHOS (vs. 0 in the opposite direction): sign test two-sided *p* = 0.000002 (i.e. strongly different from expectation by chance). Similarly, hsCRP correlates with 19 of the 20 symptoms in the predicted direction, with only one in the opposite direction: sign test *p* = 0.00004. However, as we look in greater detail at the strength of the relationship to symptoms and also at the extent to which these findings are upheld in veterans with Gulf War illness, a very different picture emerges. For CI&CIIOXPHOS, of the 20 symptoms, 17 (predicted direction) versus 0 (opposite direction) showed at least a borderline significant relation to CI&CIIOXPHOS (sign test *p* < 0.0001); and 11 of these correlations showed frank significance (that is, symptoms showed two-sided *p* < 0.05) in the predicted direction versus 0 in the opposite direction: sign test *p* < 0.001. In contrast, for hsCRP, in the total sample, only one symptom was significant or borderline significant in the predicted direction (tiredness) versus 0 in the opposite direction: sign test nonsignificant (not meaningfully testable). This may relate to the fact that a major cause of tiredness is (often undiagnosed) sleep apnea, which in turn relates strongly—as both cause and consequence—to greater body mass index/fat, and adipose tissue is a major source of inflammatory mediators.

Considering VGWI cases separately, the sign of relationship of CI&CIIOXPHOS was in the predicted direction for 18 out of 20 symptoms versus two in the opposite direction, highly unlikely to be attributable to chance: sign test *p* < 0.001. The smaller sample size reduced power for individual symptoms to attain significance. Nonetheless, six were of at least borderline significance in the predicted direction vs. none in the opposite direction: sign test *p* = 0.031. In contrast, for hsCRP, more symptoms than not showed the “wrong” direction relationship within VGWI separately (seven predicted direction vs. 13 opposite direction, nonsignificant on sign test). Moreover, in affected Gulf War veterans assessed separately, only one symptom correlated to hsCRP with significance or borderline significance, and the relationship was in the direction *opposite to prediction*: Greater inflammation was tied to *lesser* problems with the (cognitive) symptom. Thus, evidence supports a far more robust relation of the mitochondrial measure to GWI-relevant symptoms, in the overall sample and in VGWI separately, than does inflammation—which appears entirely unrelated to GWI symptoms within VGWI themselves.

Tables 6 and 7 show the central analysis, evaluating the relationship of CI&CIIOXPHOS and of hsCRP to overall summed symptom severity (indexed by the summed 20-item symptom score) assessed via regression with robust standard errors, considering the mitochondrial and inflammatory variables as predictors in models separately and together, in the total sample and in VGWI (cases) separately. Lower mitochondrial CI&CIIOXPHOS significantly predicted greater summed GWI symptoms in all four assessments, in each case with significance or strong statistical significance. In contrast, hsCRP did not positively predict symptoms in any of the four analyses. It showed no apparent relationship to summed symptom severity in the total sample, with or without adjustment for the mitochondrial measure, or in VGWI separately in the analysis unadjusted for the mitochondrial measure. Unexpectedly, within VGWI, the *independent* relation of hsCRP (after adjusting for the mitochondrial measure) to summed (GWI) symptom severity was strongly significant, but in the opposite direction to that which has long been hypothesized. That is, greater inflammation after controlling for the mitochondrial measure was significantly tied to *less* GWI symptom severity. (Addition of BMI adjustment, see Table 7 legend, did not materially affect any of these findings.)

**Table 6:** Summed symptom severity (GWI severity): single predictor models.

Predictor	Total sample			GWI case		
	β	SE	*P*	β	SE	*P*
CI&CIIOXPHOS	− 1.10	0.25	**< 0.001**	− 0.85	0.33	**0.020**
HsCRP	4.33	3.27	0.19	− 1.43	1.70	0.41

Significant *p*-values are in bold.

**Table 7:** Summed symptom severity (GWI severity): combined model.

Predictor	Total sample			GWI case		
	β	SE	*P*	β	SE	*P*
CI&CIIOXPHOS	− 1.04	0.28	**0.001**	− 1.05	0.34	**0.007**
HsCRP	1.72	2.53	0.50	− 3.80	0.78	**< 0.001**

Adjustment for BMI as a potential source of inflammation that can be elevated for VGWI^6,8,45^ did not materially affect findings for either VGWI or for the total sample. Total sample, CI&CIIOXPHOS: β = − 0.97, SE = 0.31, *p* = 0.004. HsCRP: β = 1.40, SE = 2.48, *p* = 0.58. BMI: β = 3.15, SE = 3.28, *p* = 0.35. In Gulf War veterans separately, CI&CIIOXPHOS: β = − 1.01, SE = 0.27, *p* = 0.002. HsCRP: β = − 3.63, SE = 0.73, *p* < 0.001. BMI: β = 4.14, SE = 3.91, *p* = 0.31. BMI showed a non-significant positive relationship to hsCRP in the total sample; however, the coefficient was negative and did not approach significance in VGWI separately, suggesting that BMI is not an important contributor to inflammation (indexed by hsCRP) in VGWI. Significant *p*-values are in bold.

Supplement Figs. 1 and 2 show scatterplot values of GWI severity (summed symptom severity) vs CI&CIIOXPHOS in the full sample (Supplement Fig. 1) and in VGWI separately (Supplement Fig. 2).

## Discussion

This is the first study to directly assess mitochondrial respiratory chain function on muscle biopsy in VGWI—and to assess it together with inflammation. Findings suggest a radical rethinking of the respective roles of mitochondrial impairment and inflammation in this condition. While both bioenergetic impairment and elevated (peripheral) inflammation discriminate VGWI in prior studies and also show apparent case–control differences in this study, it is seen here for the first time that the two correlate closely to one another in VGWI but not in controls—but also, for the first time, that despite this correlation, only mitochondrial impairment relates either to individual GWI symptoms or to overall GWI-relevant symptom severity. To restate, although elevated hsCRP correlates to mitochondrial impairment in VGWI, elevated hsCRP does not relate positively to GWI symptoms individually, or to GWI severity overall. When adjusted for the mitochondrial predictor, an independent association of hsCRP was unmasked (confined to VGWI)—but, unexpectedly, more elevated hsCRP was significantly tied to *less* GWI severity.

The mitochondrial finding has internal support, with a relation to symptom severity of high significance in the total sample and in VGWI separately; and both for univariable prediction and in regression adjusted for hsCRP. The hsCRP finding has high novelty, and the relation of mitochondrial indices to hsCRP selectively in VGWI, yet the opposite direction independent relation to symptoms, is intriguing and suggestive of mechanisms supported by other evidence.

The mitochondrial findings are not only internally supported but supported by external evidence. Data show that organophosphates depress signaling for fatty acid oxidation^46^, and depress enzymes critical for fatty acid oxidation^47^: Organophosphates are a key GWI-relevant exposure believed to be causal^20,48^ and used in many GWI animal models^49–56^. In fact, consistent with evidence suggesting that pesticides beyond organophosphates (including pyrethroids, organochlorines) may be implicated in GWI^48^, multiple pesticide classes share metabolomic signatures consistent with fatty acid oxidation involvement^57^. Gulf War illness animal model muscle biopsy data show a reduction in succinate dehydrogenase activity (used as an index of complex II activity)^36^.

Another finding is that the relation of select other mitochondrial indices to fatty acid oxidation indices is markedly strengthened in VGWI, consistent with yoked depressions in these indices in GWI. It is fatty acid oxidation indices selectively that relate potently to elevated hsCRP, consistent with prior evidence that depressed fatty acid oxidation promotes apoptosis^58–65^; and apoptosis promotes inflammation^41^. We hypothesize, therefore, that the increased inflammation observed in VGWI is a consequence of apoptosis (promoted by reduced fatty acid oxidation), which may serve an adaptive function, clearing impaired cells that are a drain on energy while not contributing favorably to function. This would account both for the specific correlation of inflammation to fatty acid oxidation indices in VGWI, and for the *favorable* independent association of inflammation (after accounting for mitochondrial impairment) to GWI severity.

Also relevant to the yoked depression in mitochondrial indices is the observation that depression in different mitochondrial indices relate differentially to GWI symptoms: A mitochondrial measure reliant on sugar bears the strongest relation to cognitive indices (consistent with the knowledge that the brain, at ~ 2% of the body weight, uses ~ 50% of the total body glucose^66^); while a fatty acid oxidation measure related most strongly to muscle-energetics relevant symptoms (low energy, muscle weakness, fatigue with exertion), consistent with the important role of fatty acid oxidation in muscle energetics and indeed the primacy of its role for resting muscle^67,68^. Yet a different mitochondrial measure related most strongly to cold limbs (CIIMaxUC). This measure relates to uncoupled energy production (i.e. in the presence of an uncoupler), the foundation for thermogenesis^69^. The fit to expectation of these relationships adds further triangulating support for study findings.

Findings need not preclude a causal role for inflammation in conditions known to be elevated in GWI (like cardiovascular disease): However, mitochondrial impairment could also explain these relations given that myocardial infarction, congestive heart failure, and stroke each arise from inadequate cell energy; and mitochondrial supportive supplements (which have also benefited GWI^9^, as below) have shown significant protection for each of these conditions in randomized placebo-controlled trials^70–74^.

A role for *neuro*inflammation separately was not assessed—so is neither supported nor precluded by these findings. However, findings comport powerfully with a primary role for mitochondrial impairment as an underlying basis for GWI. Moreover, the observation of elevated (peripheral) CRP helped drive the inflammatory hypothesis of GWI^28,29,75,76^. (Of note, apoptosis triggers both inflammation and also coagulation activation^41^; both inflammation and coagulation activation are reportedly elevated in VGWI^29,75^).

Our findings are consistent with others’ findings documenting increased inflammation in VGWI relative to controls, although the implications proposed are different. We do not see a relationship between inflammation and symptom severity in VGWI. We have identified only one study that reported a relationship between inflammation and symptom severity within veterans with "Gulf War illness"^76^, but this can be readily understood based on case-selection practices in that study relative to this one. We restricted to veterans that met both CDC and Kansas criteria for VGWI, to enhance specificity of the sample. (Kansas criteria are far more specific^4^). Furthermore, we excluded late-onset GWI, because there are no data on whether late-onset development of symptoms occurs more often in Gulf-deployed or whether it relates to GWI mechanisms. In contrast, the James et al. study did not exclude late-onset GWI, and accepted in their “GWI” category individuals who met either the non-specific CDC criteria or the more specific Kansas criteria.

Already within a decade after the Gulf conflict, when Era and deployed veterans were still relatively young, studies reported that 33% and 36% of Era *non-Gulf* deployed personnel met CDC symptom criteria^12,77^, and with 20 further years of aging, it may be expected that a materially higher fraction would now do so. (CDC criteria merely require that any symptom, however mild, be present for the last six months, in two of the three categories of fatigue/sleep, mood/cognitive and musculoskeletal.) Thus, early data already found that more than half of Gulf War veterans meeting these (CDC) criteria would be expected to do so irrespective of whether they had been deployed to the Gulf (with corresponding symptoms thus not attributable to Gulf exposures and associated mechanisms), and the fraction may be expected to be higher now, when veterans are older. Thus, Gulf-independent conditions and mechanisms, including inflammatory conditions leading to symptoms, would be expected to be present in more than half the veterans selected for that study’s sample. This could readily produce the appearance of a connection between inflammation and symptoms, arising from the potentially-majority fraction of participants whose symptoms were not attributable to their Gulf-deployment. (Future studies could examine the relation of inflammation and of mitochondrial impairment to symptoms in those with early onset symptoms who fully meet Kansas (and CDC) Gulf War illness criteria, compared to those who meet CDC but not Kansas criteria; and in those with late-onset GWI. Whether late-onset symptom accrual in deployed personnel differs from that in non-deployed and legitimately relates to GWI mechanisms is unknown.)

Mitochondrial impairment is a fit for all of the distinctive features of GWI, and indeed it is this that led us to first examine a role for mitochondrial impairment in this condition. Gulf War illness is characterized by high symptom multiplicity within individuals^7,78^ and high symptom variability across individuals^7,78^, consistent with mitochondrial impairment^79–81^. While symptoms are protean and traverse many domains, there is a focus on fatigue as well as brain and muscle symptoms^8^—i.e. a preferential effect on post-mitotic organs with high energy demand, as expected in mitochondrial impairment^82–87^. Variable latency to symptom onset (for veterans, following Gulf theater exposures^88^) is a well-characterized feature in mitochondrial illness, even extending to heritable mitochondrial conditions in a single kindred^89^. This variable latency is recognized to arise from the concurrent mitochondrial constructs of heteroplasmy (variability in mitochondrial compromise within and across tissues within an individual, differing across individuals) and clinical threshold effects^90–92^ (in which a certain degree of mitochondrial impairment and/or resulting cell loss is required for energetic or cellular reserves to be overcome and symptoms to emerge). Magnification of symptoms and defects following exercise in VGWI is also a fit^93^—as energetic demand would more decisively surpass supply in that setting, and as needed energy substrates are diverted from other tissues to strive to meet heightened energy needs of exercised muscle. Also consistent with mitochondrial impairment is the worsening (often reported by VGWI) of symptoms and impairments following exposure to any of a wide array of drugs and chemicals that increase oxidative stress or mitochondrial impairment. (Many drugs and chemicals, irrespective of their nominal specific mechanism of action, confer much or most of their toxicity through mitochondrial and oxidative stress mechanisms^2,94–112^.) Mitochondrial impairment is a leading hypothesis for aging^83,91,113–118^, and the resemblance of GWI to accelerated aging has been noted, by us as well as by others^16^—including increases in aging-related conditions like cardiovascular disease (tied to impaired energy and alleviated by mitochondrial supports^70–72,119^); and metabolic syndrome factors like hypertension (tied to impaired energy^120^ and alleviated by mitochondrial supports^121^).

Some inflammatory mediators have been reported to increase fatty acid oxidation^59^, which could conceivably represent a further mechanism by which increased inflammation may be tied to lesser GWI severity, after mitochondrial impairment is considered. This could conceivably further contribute to the observed independent association of inflammation to lesser GWI severity, after accounting for mitochondrial impairment (indexed by CI&CIIOXPHOS). Findings may or may not relate to reports of symptom improvement with infection/inflammatory state in a subset of patients with mitochondrial-related disorders such as autism spectrum disorder^122,123^ (in which a subset show fatty acid oxidation defects^124^, and where fever has been tied to improved symptoms in a subset^125,126^).

We have previously suggested apoptosis as a route to inflammation in GWI^9,127,128^. Data here, showing a strong correlation between fatty acid oxidation defects and increased inflammation specific to VGWI, comport with this hypothesis: as above, fatty acid oxidation has been reported to protect against apoptosis in cell types where this has been examined^58–65^. Low fatty acid oxidation in VGWI leads to loss of apoptosis protection by this mechanism, resulting in increased cell death and consequent inflammation. Further consistent with this hypothesis, reduced fatty acid oxidation has been tied to increased ceramide synthesis^58^: increased ceramides were the most striking metabolomic alteration that separated VGWI from controls in our prior metabolomic study^128^. This is relevant because ceramides are pivotal drivers of mitochondrial apoptosis^129^.

Additional evidence favoring apoptosis as a feature in GWI comes from prostaglandin findings. Prostaglandin F2α, an eicosanoid known to protect against myocyte apoptosis^130^, was found to be strongly and significantly depressed in VGWI^127^. The degree of depression related strongly and significantly to the degree of self-rated muscle weakness^127^. (Self-rated muscle weakness has been validated against actual physical function^9^, and as in the methods, has also been shown to relate significantly to mitochondrial/bioenergetic impairment in VGWI assessed by post-exercise phosphocreatine recovery time constant determined on ^31^Phosphorus Magnetic Resonance Spectroscopy. Phosphocreatine is a backup muscle energy source that is depressed with exercise and the rate of recovery is known to relate to the rate of ATP production^131^). Though significantly depressed in VGWI, prostaglandin F2α did *not* relate to overall GWI symptom severity, just as we find here to be the case for inflammation. As above, the apparent protective association of hsCRP to GWI severity in VGWI selectively (after accounting for mitochondrial CI&CIIOXPHOS) could be compatible with a *protective* role of apoptosis against some symptoms. Of course, ongoing apoptosis in tissues with poor regenerative potential (like brain and to a lesser extent muscle) can, over time, cause problems—and brain atrophy is observed in VGWI^93,132^. If the mechanisms proposed here are operative, interventions should be prioritized for testing that support mitochondrial function/integrity and *thereby* limit “need” (and drive) for such apoptosis. Examples include coenzyme Q10 (ubiquinone) which in fact was reported to benefit symptoms and function in VGWI, in a double-blind RCT^9^. Other agents with reported tentative benefit (small placebo-controlled crossover study) such as curcumin and resveratrol^133,134^, though reportedly chosen for their anti-inflammatory properties, may alternatively have conferred relief in GWI through their documented benefits to mitochondrial status including fatty acid oxidation^124,135–144^. (Where alleviation of inflammation is produced through benefit to impaired mitochondrial function, clinical benefit would be expected under the mitochondrial-impairment hypothesis for GWI. Indeed, treatments that benefit impaired mitochondrial status may be expected to improve clinical outcomes, whether or not inflammation is reduced through a distinct pathway^145^).

As a secondary comment meriting follow-up, we note that sleep was the one symptom that did not, in the prior coenzyme Q10 study, show the favorable direction association with coenzyme Q10 versus placebo (the relation was almost exactly neutral)—and that here it was one of two symptoms not showing the predicted direction association to CI&CIIOXPHOS in VGWI. In the total sample, moreover, “tiredness” (which is commonly related to sleep problems) related significantly to inflammation—although no relation was observed in VGWI separately.

Given apparent trends toward lesser mitochondrial function, possibly spanning mitochondrial measures, reduced mitochondrial content (or mitochondrial DNA) could be one contributor. (However, while correlations among some mitochondrial indices appear more positive in VGWI than in controls, consistent with coordinated shifts, this is not a consistent finding across all indices). Future studies can assess this and other potential foundations for the observed findings.

The study is of modest size, but it is desirable to keep sample size to a minimum for a study employing an invasive procedure. Despite the modest size sample, a strongly significant relation between depressed mitochondrial function and increased GWI severity was observed, underscoring the large effect size. This study did not assess markers of inflammation besides hsCRP, which could bear different relations to GWI. However, the previously reported relation of increased CRP to GWI contributed to the dominance of the inflammatory hypothesis of GWI^28,29,75,76^, a hypothesis that the present findings appear to controvert. Regarding non-CRP inflammatory markers, studies have shown inconsistent findings: For instance, one study reported increased IL-4 and IL-13^146^ and another study reported decreased IL-4 and IL-13 in GWI^147^. Findings do not necessarily preclude a role for inflammation in some conditions noted to be elevated in GWI. Moreover, this study does not assess *neuro*inflammation, or its relation to mitochondrial impairment or to symptoms.

GWI is an exemplar of a growing cadre of environmentally-triggered chronic multisymptom illnesses, most if not all of which share at their core exposure to mitochondrially toxic agents, rendering findings in Gulf War veterans of broader relevance. Illustrative of the broad scope of mitochondrially toxic factors that have been tied to chronic multisymptom illness are organophosphate pesticides^3,148–150^, fluroquinolones^2,151^, carbon monoxide^152–154^, (long) Covid^155–157^ and non-ionizing radiation toxicity including so-called “Havana Syndrome”^1,158^. Both organophosphates and fluroquinolones are among implicated agents in GWI, that bear relevance to civilian and other military settings. Thus, our findings in GWI have potential relevance to chronic multisymptom fatiguing illness outside of GWI, both due to agents implicated in GWI, and other agents bearing shared mechanisms of toxicity. Additionally, these findings may have implications for aging—and may prove relevant to interpreting (or reinterpreting) the basis of the relation of inflammation to other health conditions. (For instance, inflammation is tied to cardiovascular disease—but anti-inflammatory agents promote rather than prevent cardiovascular events.)

To summarize, for the first time, direct muscle biopsy data affirm the presence of mitochondrial impairment in VGWI, specify the character of that compromise, and document a powerful relation of mitochondrial compromise to GWI severity. For the first time, data document a strong relation of mitochondrial impairment to inflammation in VGWI selectively, and clarify that mitochondrial impairment but not inflammation positively predicts GWI symptoms in VGWI. Indeed, we document an apparent protective association of inflammation to GWI severity in VGWI, once mitochondrial impairment is considered: This highly novel finding clearly requires replication. We speculate that the inflammation may arise from and serve as a proxy for an association to mitochondrial apoptosis, perhaps adaptively triggered—which may help to clear impaired cells. Further research is needed to affirm or refute this hypothesis.

## Materials and methods

### Ethics statement

The study protocol was approved by the University of California, San Diego, Human Research Protections Program (Project #151863) and Department of Defense Human Research Protections Office (Project #A-18779). All subjects gave written informed consent to participate. All methods were performed in accordance with relevant guidelines and regulations.

Forty-two participants included 21 veterans meeting both CDC and Kansas criteria for GWI and 21 healthy controls.

Cases: To qualify as a GWI case, screened prospective participants were required to have been deployed to the Persian Gulf theater of operations at any time from August 1, 1990 through July 31, 1991, inclusive. They were required to meet both CDC^8^ and Kansas^7^ symptom inclusion criteria for GWI. Both criteria require chronic symptoms of at least six months duration, arising during or after Gulf-theater deployment. CDC criteria require that these symptoms are present in at least two of the three domains of fatigue, mood/cognitive, and musculoskeletal. Kansas criteria are more discriminating and require symptoms in at least three of the six categories of fatigue/sleep, pain, neurological, respiratory, gastrointestinal and dermatologic. In addition, for a domain to qualify toward Kansas GWI criteria, symptoms within the domain must be at least moderate in severity (not mild) and/or multiple symptoms must be present in the category.

Exclusions encompass: (1) concurrent conditions (like lupus or multiple sclerosis) that can cause symptoms that might be confused with GWI; (2) general self-rated health prior to the Gulf War (cases) or in 1990 (controls) retrospectively rated as less than “very good” or “excellent” (in order to exclude those that might have had other processes affecting their health already present at that time); (3) late onset symptoms, on grounds that GWI criteria were only shown to be discriminating in the early time period after the Gulf War (tapping ~ early onset symptoms in primarily young veterans) and accrual of symptoms with extended time and aging might spuriously cause some individuals to meet GWI criteria in whom GWI processes are not involved. It is unknown whether gradual accrual of symptoms encompassing several domains in the many years following the Gulf War as participants age need necessarily relate to GWI, to Gulf War deployment, or to GWI processes. That is, GWI case criteria have not been validated for those with late-onset GWI. At about one fourth through study enrollment, a requirement was added that cases must have at least moderate muscle weakness, to address mitochondrial “heteroplasmy” (in which there can be variable involvement of mitochondrial impairment across organs, necessitating matching of the organ assessed to an organ with clinical compromise^159^). It is understood to be preferable to sample an affected tissue if assessing for mitochondrial impairment^90–92^. Conversely, if sampling a specific tissue, participants symptomatic in that tissue should be the focus.

Controls: To qualify as a control, participants were required to be non-veterans (to exclude those with a *forme fruste* of GWI, since evidence suggests that some veterans continue to newly meet criteria for GWI years later, and it is unknown whether these too in fact have GWI). Controls were required to meet neither CDC nor Kansas symptom criteria. To be eligible as a control, participants must have had a maximum of one symptom of at least moderate severity or two “mild” symptoms. Exceptions were considered in selected circumstances. For instance, if an individual had significant joint pain related to a discrete injury, that was clearly not related to systemic processes, this need not be exclusionary. Controls were also required not to have any Kansas exclusionary condition (like lupus or multiple sclerosis). Controls were selected to match 1:1 to a qualified case on age (within 5 years), sex, and race/ethnicity. (Half matches on ethnicity were accepted in recognition of the reality of mixed ethnicities.)

Because headache is not uncommonly tied to mitochondrial/bioenergetic impairment or energy supply/demand mismatch^160–164^, prospective controls who reported headache were excluded.

Assessments: Each participant underwent phlebotomy on the same day as the muscle biopsy.

HsCRP, a measure of peripheral inflammation, was assessed at the UCSD clinical laboratory using the latex-enhanced immunoturbidimetric method^165^. HsCRP is preferred over CRP for the range 0.5–10 mg/L (with CRP preferred for the range 10–1000 mg/L). Other studies have reported increased CRP in VGWI relative to selected healthy controls, but have noted that most values are in the normal range, usually defined as < 3 mg/L. Therefore, hsCRP was chosen as the preferred metric for inflammation with superior validity and sensitivity within the expected range.

Questionnaires: Participants completed Qualtrics surveys providing information on demographics, military history, exposure history, and symptoms/GWI severity.

Summed symptom severity: The UCSD GWI Symptom Survey was administered, assessing 20 symptoms each found to be present in ≥ 50% of VGWI in a prior study^9^. Severity of each symptom over the prior two weeks was rated 0–10.

This symptom survey has undergone validation as have a number of the individual symptom queries within the survey. The summed symptom score shows convergent validity, strongly correlating with the summed score from Kansas GWI criteria in a mixed sample of VGWI and controls (r = 0.91, *p* < 0.0001) and general self-rated health by visual analog scale (r = − 0.75, *p* < 0.0001).

Muscle weakness assessment: A muscle weakness self-rating of > 5 (out of 10) defined muscle weakness that was of at least moderate severity. The single item self-rating of muscle weakness (as above a qualifying criterion for GWI cases) was previously validated against objectively measured physical function assessed by the lower extremity Summary Performance Score^166^ (r = − 0.47, *p* = 0.001) procured in Gulf War veterans in a previous study based on averages of duplicated measurements of each (assessed ~ 1 week apart)^9^; and found to relate to bioenergetics assessed by post-exercise phosphocreatine recovery time constant (PCr-R) on ^31^Phosphorus Magnetic Resonance Spectroscopy: muscle weakness rating 0–5, PCr-R mean (SD) = 31.1 (6.95); muscle weakness rating 6–10, PCr-R mean (SD) = 45.4 (13.0), difference *p* = 0.01. It has also been shown to be sensitive to change with (double-blinded) treatment with an agent with potential to adversely affect muscle^167^.

Percutaneous skeletal muscle biopsy was performed (by co-I Taub) using the modified Bergström technique^168^ at the UCSD Altman Clinical and Translational Research Institute with a nurse experienced in assisting in the biopsy procedure. A single muscle biopsy was performed for each participant, and all mitochondrial assessments were conducted on the resulting sample. No more than one biopsy was performed on a given day. Participants were instructed not to take blood thinning agents (such as aspirin, nonsteroidal anti-inflammatory agents, clopidogrel) for 7 days prior to the procedure. Participants were prepped for the procedure using the Universal Protocol Checklist. The procedure field was sterilized with chlorhexidine and the operator employed hand hygiene, a protective gown, and sterile gloves. A partial body drape was used to keep the biopsy field sterile and a ~ 20 cm diameter area proximal to the knee joint was cleaned three times with chlorhexidine and dried. A 1 cm area in the center of the sterilized field was anesthetized twice with ~ 10 cc of 1% lidocaine, first subcutaneously, then intramuscularly into the vastus lateralis muscle. After waiting ~ 5 min for the anesthetic to take effect, an incision was made to the depth of a #11 scalpel blade. Using a Bergström needle, one rapid pass was made while suction was being provided to the inner trochar into the vastus lateralis muscle, retrieving approximately 150–220 mg of muscle.

Mitochondrial assessment was conducted under the supervision of co-I Patel, using three published protocols^169^. The first was fatty acids with sequential substrates, the second was MiR05 (mitochondrial respiration medium) with sequential substrates, and the final was MiR05 with rotenone and complex II substrates only. Fresh muscle biopsies from subjects were placed directly in ice-cold BIOPS solution. (The muscle biopsy was dissected, and 9 pieces of muscle fiber were put into 9 separate ports. Groups of 3 ports then underwent coupled (CI through CII or CII directly with CI inhibition) assays with fatty acids or sugar as substrates.) BIOPS, a medium designed to relax and preserve muscle biopsy samples, comprised 10 mM Ca-EGTA buffer, 0.1 µM free calcium, 20 mM imidazole, 20 mM taurine, 50 mM K-MES (2-(*N*-morpholino)ethanesulfonic acid, a buffering agent), 0.5 mM dithiothreitol, 6.56 mM MgCl2, 5.77 mM ATP, 15 mM phosphocreatine, pH 7.1. After transporting the samples to the laboratory, skeletal muscle fibers were manually dissected under a microscope in BIOPS and then incubated with saponin (50 mg/mL) in BIOPS for 20 min. Then the sample was equilibrated in MiR05 respiratory buffer (110 mM D-sucrose, 60 mM lactobobionic acid, 20 mM taurine, 10 mM KH2PO4, 3 mM MgCl2, 20 mM HEPES, 0.5 mM EGTA, and 0.1 mg/mL bovine serum albumin at pH 7.1) at 37 °C for 5 min. Tissue weight was obtained after lightly patting with weighing paper, and 0.6–1.2 mg of muscle fiber was added to each chamber of an Oroboros O2k high-resolution respirometry system containing MiR05 respiratory buffer. Responses and changes in oxygen flux were normalized to mass of tissue in the chamber. Before addition of tissue, O_2_ was injected in each Oxygraph-2k chamber and equilibration of the gas phase with MiR05 was set to 400–450 µM before addition of muscle fibers. O_2_ flux measurements were typically made with 200–400 µM O_2_. The chamber was allowed to equilibrate for 10 min after addition of muscle fibers. After equilibration, coupled pathway assessments were made with and without fatty acids (octanoylcarnitine, [0.8 mM]) through mitochondrial complexes (“C”) by assessing leak (CI substrates glutamate [10 mM]/malate [2 mM], “L”), oxidative phosphorylation (ADP [1.25 mM] added, “Ox”), followed by addition of CI substrate pyruvate [5 mM] (“CI Ox”), then CII substrate succinate [10 mM] (CI&CII Ox), then carbonilcyanide p-trifluoromethoxyphenylhydrazone (FCCP, [0.5 µM] steps until max uncoupling) to uncouple mitochondria (mUC), then addition of rotenone [0.5 µM] to inhibit CI to assess CII uncoupled (“CII ETS”), and antimycin A [2.5 µM] to inhibit CII and CIII activity to assess residual oxygen consumption (“ROX”). CII coupled respiration was assessed without other substrates. Oxygen flux trace and rate determinations were obtained using DatLab 7 software. Cytochrome c [10 µM] was added after ADP to assess mitochondrial quality. The preparation was not utilized if respiration increased greater than 10% after addition of cytochrome c. All assessments were performed in technical triplicate.

As per the Introduction, based on preliminary analyses, four indices of mitochondrial function are highlighted in this study. (To ease understanding of the abbreviations based on component, CI and CII refer to mitochondrial complexes I and II, respectively; FAO refers to fatty acid oxidation; ETS refers to respiratory capacity of the electron transport system: MaxUC refers to the uncoupled state.) CI&CII&FAOCI/CIIOXPHOS refers to assessment of that aspect of mitochondrial function determined by using fatty acids as the fuel source with complex I and II substrates malate, glutamate, pyruvate, succinate and adenosine diphosphate ADP to drive respiration. CII&FAOETS refers to assessment of that aspect of mitochondrial function determined by using fatty acids as the fuel source with rotenone to inhibit complex I to look at complex II respiration. FAOMaxUC refers to assessment of that aspect of mitochondrial function determined by using fatty acids as the fuel source with FCCP as an uncoupler. CI&CIIOXPHOS refers to assessment of that aspect of mitochondrial function determined by using glucose as the fuel source with complex I and II substrates malate, glutamate, pyruvate, succinate with ADP to drive respiration.

Blinding of mitochondrial assessments: All mitochondrial assessments were conducted blinded to case–control status.

Analysis:

I. Descriptive statistics were assessed for participant characteristics, for all participants and for VGWI and controls separately. These included means (standard deviations) for continuous variables and proportions for categorical variables. Separate analyses were performed for matched case–control pairs, and for the expanded sample who contributed to the primary cross-sectional analyses of relations of mitochondrial and inflammatory measures to outcomes. *T*-tests of difference in means (continuous variables) and chi-square tests (categorical variables) assessed case–control differences; however, case–control comparisons are not the focus of the present analyses, which are directed to prediction of summed symptom severity.
II. Case–control differences in important candidate mitochondrial markers and in hsCRP were assessed in matched pairs (paired *t*-tests) and in the full sample (unpaired *t*-tests). Because of prior evidence of impaired mitochondrial function and increased hsCRP in VGWI, for this peripheral assessment, one-sided *p*-values may be justified, and are mentioned (legend only) to assist in determining whether the present study is concordant with prior findings showing elevated inflammation and mitochondrial impairment in GWI; and in helping to characterize the nature of mitochondrial respiratory chain impairment.
III. The interrelation of mitochondrial markers to one another and to inflammation was provisionally examined via correlation of candidate mitochondrial markers and hsCRP to one another, using Pearson’s correlation coefficients.
IV. Correlations of candidate mitochondrial markers and of hsCRP to each of the twenty symptoms of the UCSD GWI symptom survey were also assessed in all participants, and separately in cases and controls. This focused on the mitochondrial marker of interest that showed the most dissociation from hsCRP, to provide better discrimination (to reduce collinearity).
V. Sign tests were employed to assess whether the sign of the correlation was more often in the predicted than the converse direction (more mitochondrial impairment or inflammation tied to greater symptoms is predicted for symptoms overall), as well as considering those symptoms with at least borderline significant correlations (two-sided *p* < 0.1), and those with a frankly significant correlation (two-sided *p* < 0.05) considering mitochondrial indices of relevance and hsCRP. Although the sign test is simple, it has critical advantages: It makes no distributional assumptions and carries high authority. Indeed, statistics experts state that “if the Sign-test indicates a significant difference, and another test does not, you should seriously rethink whether the other test is valid”^170^.
VI. Summed symptom severity in the total sample and within VGWI (cases) was assessed via regression analysis with robust (heteroskedasticity-independent) standard errors^171^, examining univariable prediction by the chosen mitochondrial marker and hsCRP, then in joint analysis.
VII. Study power: The originally proposed sample size of 54 provided 80% power with two-sided alpha of 0.05 to detect a difference between cases and controls of 0.4 standard deviations. Shutdown of the study due to Covid-19, and enforced extended delays in study resumption due to the requirement for sustained close proximity of multiple parties for the biopsy procedure, led the study to be suspended prior to initial recruitment targets being achieved. Findings were deemed sufficiently striking that the determination was made not to subject additional individuals to the invasive muscle biopsy procedure. For the paired sample, with the achieved sample size, there was 80% power with two-sided alpha of 0.05 to detect an effect size of 0.56 standard deviations. However, a focus on the relation of mitochondrial indices to summed symptom severity rather than dichotomous outcomes more strongly supports study power by capitalizing on the continuous nature of both the mechanistic (mitochondrial and inflammatory) and summed symptom severity variables.
VIII. All statistical analyses employed Stata® (versions 8.0 and 13.0, College Station, Texas). P-values are two-sided except where otherwise stated. *P*-values < 0.05 designated statistical significance. Adjustment for multiple comparisons rests on the presumption that chance is the first order explanation for significance of findings, if observed^172^. This presumption is obviated given past evidence of bioenergetic/mitochondrial impairment in VGWI, as well as in animal models of GWI, and multiple other features of GWI that comport with known features of mitochondrial illness (see Discussion). Thus, no adjustment for multiple comparisons was undertaken.

## Supplementary Information

Supplementary Information 1.

## Data Availability

Data will be made available on request. Participants were advised that their data would be kept confidential, with only group-level data published. Because specific demographic and symptom information could potentially identify individual veterans, we prefer to share the data on an as-requested basis, with assurances of confidentiality protections, rather than in a public-use database. Requests for data should be made to Dr. Golomb (bgolomb@ucsd.edu).
