# Supplementary material for: Mitochondrial impairment but not peripheral inflammation predicts greater Gulf War illness severity
Source: Sci Rep. 2023 Jul 12;13:10739. doi: 10.1038/s41598-023-35896-w (PMC10338554; doi:10.1038/s41598-023-35896-w)
Supplement: Supplementary file 1 — Supplementary Information 1. [file 41598_2023_35896_MOESM1_ESM.docx]

**Mitochondrial impairment but not peripheral inflammation predicts greater Gulf War illness severity**

Beatrice A. Golomb, MD, PhD^1^*, Roel Sanchez Baez, MD^1^,^2^, Jan M. Schilling, MD^3^, Mehul Dhanani, BS^3,4^, McKenzie J. Fannon, BS^3^, Brinton K. Berg, BA^1^, Bruce J Miller, BS^1^, Pam R Taub, MD^5^ and Hemal H. Patel, PhD^3^

1. Department of Medicine, University of California, San Diego, La Jolla, CA 92093, U.S.A.
2. Current Affiliation: San Ysidro Health Center, San Diego, CA 92114, U.S.A.
3. VA San Diego Healthcare System and Department of Anesthesiology, University of California, San Diego, San Diego, CA 92161, U.S.A.
4. Current Affiliation: Avidity Biosciences, San Diego, CA 92121, U.S.A.
5. Division of Cardiovascular Medicine, Department of Medicine, University of California, San Diego, San Diego, CA 92037, U.S.A.

***Corresponding Author**:

Beatrice Alexandra Golomb, MD, PhD

Department of Medicine

UC San Diego School of Medicine

9500 Gilman Drive #0995

La Jolla, CA 92093-0995 U.S.A

**Email:** [bgolomb@ucsd.edu](mailto:bgolomb@ucsd.edu)

| **Supplement Table 1.** All mitochondrial assessments by case-control status. | | | | | | | | | | |
| --- | --- | --- | --- | --- | --- | --- | --- | --- | --- | --- |
|  | **Total Sample (N=36)**  **Mean**  **(SD)** | **Unpaired Analysis** | | | | **Paired Analysis** | | | | **Description** |
|  |  | **VGWI**  **(n=19)**  **Mean**  **(SD)** | **Controls**  **(n=17)**  **Mean**  **(SD)** | **Difference**  **Mean** | **P*** | **VGWI**  **(n=19)**  **Mean**  **(SD)** | **Controls**  **(n=17)**  **Mean**  **(SD)** | **Difference Mean  (SD)** | **P*** |  |
| **FAO**  **Oct&G&M**  **Leak** | 7.37  (8.91) | 6.69  (7.31) | 8.14  (10.7) | 1.45 | 0.66 | 8.57  (6.73) | 10.2  (12.2) | 1.66  (15.1) | 0.74 | Determined by using fatty acids as the fuel source with complex I substrates malate/glutamate in the absence of ADP to assess leak. |
|  |  |  |  |  | 0.33 |  |  |  | 0.37 |  |
| **CI&FAO**  **G&M**  **OXPHOS** | 24.1  (12.5) | 25.2  (9.11) | 22.8  (15.8) | -2.35 | 0.62 | 24.1  (8.96) | 28.2  (14.1) | 4.12  (16.7) | 0.45 | Determined by using fatty acids as the fuel source with complex I substrates malate/glutamate with ADP to drive respiration. |
|  |  |  |  |  | 0.31 |  |  |  | 0.23 |  |
| **CI&FAO**  **G&M&P**  **OXPHOS** | 34.5  (20.3) | 34.2  (16.7) | 35.0  (24.4) | 0.77 | 0.92 | 27.8  (14.8) | 41.9  (24.6) | 14.1  (30.1) | 0.17 | Determined by using fatty acids as the fuel source with complex I substrates malate, glutamate, and pyruvate with ADP to drive respiration. |
|  |  |  |  |  | 0.46 |  |  |  | 0.087 |  |
| **CI&CII&**  **FAO**  **CI/**  **CII**  **OXPHOS** | 56.4  (27.1) | 50.7  (24.0) | 62.9  (29.8) | 12.2 | 0.23 | 46.0  (23.7) | 72.1  (29.6) | 26.1  (33.6) | **0.037** | Determined by using fatty acids as the fuel source with complex I and II substrates malate, glutamate, pyruvate, and succinate with ADP to drive respiration. |
|  |  |  |  |  | 0.11 |  |  |  | **0.018** |  |
| **FAO**  **MaxUC** | 82.7  (36.1) | 74.8  (29.5) | 91.9  (41.7) | 17.1 | 0.20 | 67.6  (26.7) | 102.1  (45.5) | 34.4  (47.9) | **0.049** | Determined by using fatty acids as the fuel source with FCCP as an uncoupler. |
|  |  |  |  |  | 0.10 |  |  |  | **0.025** |  |
| **CII&FAO**  **ETS** | 49.4  (18.1) | 44.8  (13.7) | 54.9  (21.6) | 10.1 | 0.14 | 45.2  (15.5) | 59.1  (22.4) | 13.8  (24.1) | 0.10 | Determined by using fatty acids as the fuel source with rotenone to inhibit complex I to look at complex II respiration. |
|  |  |  |  |  | 0.068 |  |  |  | 0.051 |  |
| **FAO**  **ROX** | -5.35  (7.76) | -4.13  (4.94) | -6.75  (10.1) | -2.62 | 0.37 | -2.30  (4.38) | -6.33  (11.8) | -4.03  (12.8) | 0.35 | Determined by using fatty acids as the fuel source with antimycin A to inhibit complex III to look at residual oxygen consumption. |
|  |  |  |  |  | 0.18 |  |  |  | 0.17 |  |
| **CI**  **G&M**  **Leak** | 9.28  (11.5) | 8.51  (11.7) | 10.3  (11.6) | 1.74 | 0.68 | 7.05  (9.69) | 10.4  (10.5) | 3.32  (14.6) | 0.47 | Determined by using basal MiRO5 media with complex I substrates malate/glutamate in the absence of ADP to assess leak. |
|  |  |  |  |  | 0.34 |  |  |  | 0.23 |  |
| **CI**  **G&M**  **OXPHOS** | 36.0  (22.2) | 31.8  (14.2) | 41.4  (29.3) | 9.54 | 0.23 | 33.0  (13.0) | 49.2  (27.3) | 16.3  (30.8) | 0.11 | Determined by using basal MiRO5 media with complex I substrates malate/glutamate with ADP to drive respiration. |
|  |  |  |  |  | 0.12 |  |  |  | 0.055 |  |
| **CI**  **G&M&P**  **OXPHOS** | 32.9  (23.6) | 36.9  (22.1) | 27.7  (25.3) | -9.23 | 0.28 | 36.8  (27.0) | 30.4  (28.0) | -6.41  (35.7) | 0.56 | Determined by using basal MiRO5 media with complex I substrates malate, glutamate, and pyruvate with ADP to drive respiration. |
|  |  |  |  |  | 0.14 |  |  |  | 0.28 |  |
| **CI&CII**  **OXPHOS** | 56.2  (22.8) | 51.1  (22.8) | 62.7  (21.8) | 11.6 | 0.15 | 49.0  (19.8) | 64.8  (24.0) | 15.8  (35.0) | 0.17 | Determined by using basal MiRO5 media with complex I and II substrates malate, glutamate, pyruvate, and succinate with ADP to drive respiration. |
|  |  |  |  |  | 0.077 |  |  |  | 0.083 |  |
| **CI**  **MaxUC** | 85.6  (28.6) | 79.5  (25.3) | 93.6  (31.4) | 14.1 | 0.17 | 79.4  (24.4) | 95.2  (34.5) | 15.8  (42.1) | 0.24 | Determined by using basal MiRO5 media with FCCP as an uncoupler. |
|  |  |  |  |  | 0.085 |  |  |  | 0.12 |  |
| **CII**  **ETS** | 47.3  (20.0) | 44.2  (16.9) | 51.2  (23.4) | 6.98 | 0.33 | 45.0  (15.4) | 48.4  (25.6) | 3.36  (31.5) | 0.73 | Determined by using basal MiRO5 media with rotenone to inhibit complex I to look at complex II respiration. |
|  |  |  |  |  | 0.17 |  |  |  | 0.37 |  |
| **CI**  **ROX** | -3.52  (6.84) | -2.17  (6.25) | -5.25  (7.41) | -3.08 | 0.21 | -1.06  (6.78) | -6.20  (7.59) | -5.14  (10.6) | 0.14 | Determined by using basal MiRO5 media with antimycin A to inhibit complex III to look at residual oxygen consumption. |
|  |  |  |  |  | 0.11 |  |  |  | 0.069 |  |
| **ROT**  **Bleak** | 1.40  (6.81) | 0.41  (6.48) | 2.63  (7.25) | 2.22 | 0.39 | 0.88  (7.48) | 0.81  (6.51) | -0.07  (9.09) | 0.98 | Determined by using basal MiRO5 media with rotenone to inhibit complex I in the absence of ADP to assess baseline leak. |
|  |  |  |  |  | 0.20 |  |  |  | 0.49 |  |
| **CII**  **Leak** | 17.8  (17.3) | 14.6  (15.5) | 21.6  (19.2) | 7.0 | 0.29 | 19.1  (18.2) | 17.5  (10.9) | -1.60  (23.7) | 0.84 | Determined by using basal MiRO5 media with complex II substrate succinate in the absence of ADP to assess leak. |
|  |  |  |  |  | 0.14 |  |  |  | 0.42 |  |
| **CII**  **OXPHOS** | 70.3  (28.0) | 62.0  (14.6) | 80.5  (36.8) | 18.6 | 0.075 | 64.2  (14.4) | 73.3  (28.4) | 9.06  (36.1) | 0.45 | Determined by using basal MiRO5 media with complex II substrate succinate with ADP to drive respiration. |
|  |  |  |  |  | **0.038** |  |  |  | 0.22 |  |
| **CII**  **MaxUC** | 78.4  (30.8) | 69.8  (23.5) | 88.9  (36.1) | 19.1 | 0.098 | 74.7  (14.3) | 79.6  (24.3) | 4.90  (30.4) | 0.62 | Determined by using basal MiRO5 media with FCCP as an uncoupler. |
|  |  |  |  |  | **0.049** |  |  |  | 0.31 |  |
| **CII**  **ROX** | -6.38  (18.6) | -3.07  (9.04) | -10.5  (25.9) | -7.38 | 0.29 | -3.12  (10.7) | -14.5  (28.1) | -11.41  (30.38) | 0.27 | Determined by using basal MiRO5 media with antimycin A to inhibit complex III to look residual oxygen consumption. |
|  |  |  |  |  | 0.15 |  |  |  | 0.13 |  |
| * Two sided *p*-values are complemented by one-sided *p*-values (grayed out) to make more apparent trends in the expected/predicted direction. One-sided *p*-values have some support from prior evidence of elevated hsCRP and depressed mitochondrial function in VGWI. Subtracting mean values for cases from mean values for controls does not always yield the differences presented here because values are rounded to three significant digits. MiRO5 (with 110mM D-sucrose), C (complex), FAO (fatty acid oxidation), ADP (adenosine diphosphate), P (pyruvate), M (malate), G (glutamate), S (succinate), Oct (octanoylcarnitine), OXPHOS (oxygen consumption in the ADP-activated state), MaxUC (maximum uncoupled state), FCCP (carbonilcyanide p-trifluoromethoxyphenylhydrazone, an uncoupling agent), ETS (respiratory capacity of electron transfer system), ROX (residual oxygen consumption), B (baseline). Values for respiration are pmolO_2_*s^-1^/mg tissue. Three protocols were employed to secure the data, each reflected in PMID: 27060259. | | | | | | | | | | |

| **Supplement Table 2a.** Pairwise correlations and corresponding p-values for CI&CIIOXPHOS and symptoms in controls | | | |
| --- | --- | --- | --- |
|  | **Controls (n=14)** | | |
|  | **r** | **P** | **Sign** |
| **Aches/pains** | 0.062 | 0.83 | - |
| **Joint pain** | 0.67 | **0.0092** | - |
| **Muscle pain** | -0.18 | 0.54 | + |
| **Headache** |  |  |  |
| **Tiredness** | 0.13 | 0.66 | - |
| **Sleep problems** | 0.14 | 0.63 | - |
| **Low energy** |  |  |  |
| **Muscle weakness** |  |  |  |
| **Post-exertion fatigue** | -0.25 | 0.39 | + |
| **Irritability** | -0.045 | 0.88 | + |
| **Impatience** | -0.018 | 0.95 | + |
| **Anxiety** | -0.21 | 0.48 | + |
| **Need to recheck** | -0.21 | 0.48 | + |
| **Word/name recall** | -0.018 | 0.95 | + |
| **Concentration problems** |  |  |  |
| **Difficulty remembering** | -0.018 | 0.95 | + |
| **Reading difficulty** |  |  |  |
| **Cold limbs** |  |  |  |
| **Dry skin** |  |  |  |
| **Ringing in ears** | -0.018 | 0.95 | + |
| r = Pearson’s correlation coefficient. CI&CIIOXPHOS was secured for 14 of the 17 controls. Bolded *p*-values are significant. The sign is designated as + for relations in the predicted direction and - for relations in the opposite direction. Cells are grayed out for symptoms that were inadequately represented in controls to allow for calculation of a correlation coefficient. | | | |

| **Supplement Table 2b.** Pairwise correlations and corresponding p-values for hsCRP and symptoms | | | |
| --- | --- | --- | --- |
|  | **Controls (n=17)** | | |
|  | **R** | **P** | **Sign** |
| **Aches/pains** | -0.24 | 0.34 | - |
| **Joint pain** | -0.14 | 0.59 | - |
| **Muscle pain** | 0.20 | 0.45 | + |
| **Headache** | -0.12 | 0.64 | - |
| **Tiredness** | 0.54 | **0.026** | + |
| **Sleep problems** | 0.81 | **0.0001** | + |
| **Low energy** |  |  |  |
| **Muscle weakness** |  |  |  |
| **Post-exertion fatigue** | -0.10 | 0.70 | - |
| **Irritability** | -0.093 | 0.72 | - |
| **Impatience** | -0.082 | 0.75 | - |
| **Anxiety** | -0.11 | 0.67 | - |
| **Need to recheck** | 0.38 | 0.13 | + |
| **Word/name recall** | -0.15 | 0.58 | - |
| **Concentration problems** |  |  |  |
| **Difficulty remembering** | -0.082 | 0.75 | - |
| **Reading difficulty** |  |  |  |
| **Cold limbs** |  |  |  |
| **Dry skin** | -0.12 | 0.64 | - |
| **Ringing in ears** | -0.13 | 0.61 | - |
| r = Pearson’s correlation coefficient. Bolded *p*-values are significant. The sign is designated as + for relations in the predicted direction and - for relations in the opposite direction. Cells are grayed out for symptoms that were inadequately represented in controls to allow for calculation of a correlation coefficient. We hypothesize that the relation of sleep problems and tiredness to hsCRP in controls is driven by the relation of these to sleep apnea. Sleep apnea relates to obesity/weight gain (bidirectionally), and adipose sites produce inflammatory mediators. In addition, sleep apnea (via reoxygenation injury) promotes oxidative stress, a driver of apoptosis and thereby inflammation. | | | |

**Supplement Figure 1**. GWI severity vs. CI&CIIOXPHOS: Total sample


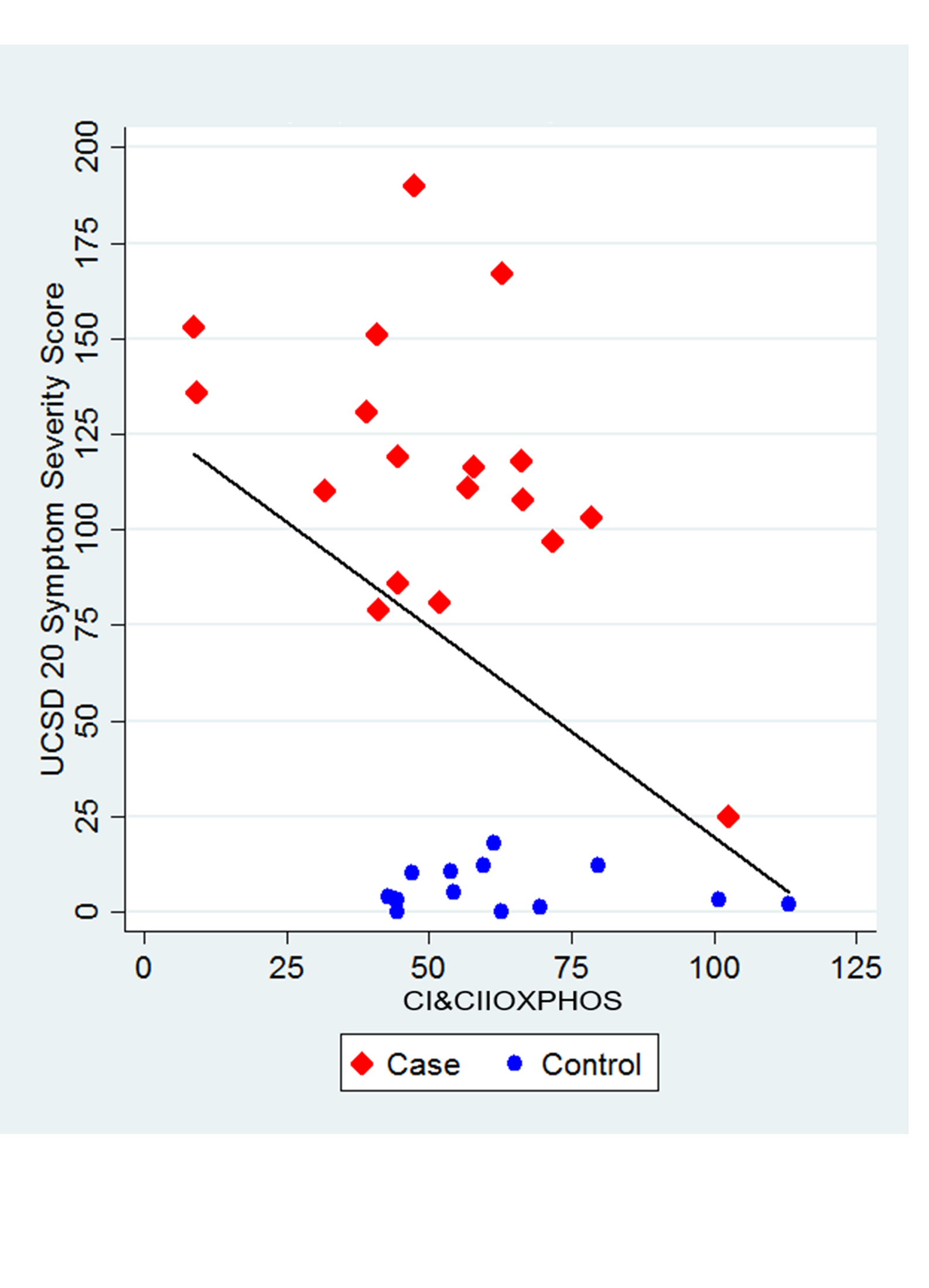


Combined Gulf War illness (case) and healthy control participants: greater symptom severity (y-axis) is tied to lower CI&CIIOXPHOS (mitochondrial function marker).

**Supplement Figure 2.** GWI severity vs. CI&CIIOXPHOS: GWI cases separately

**
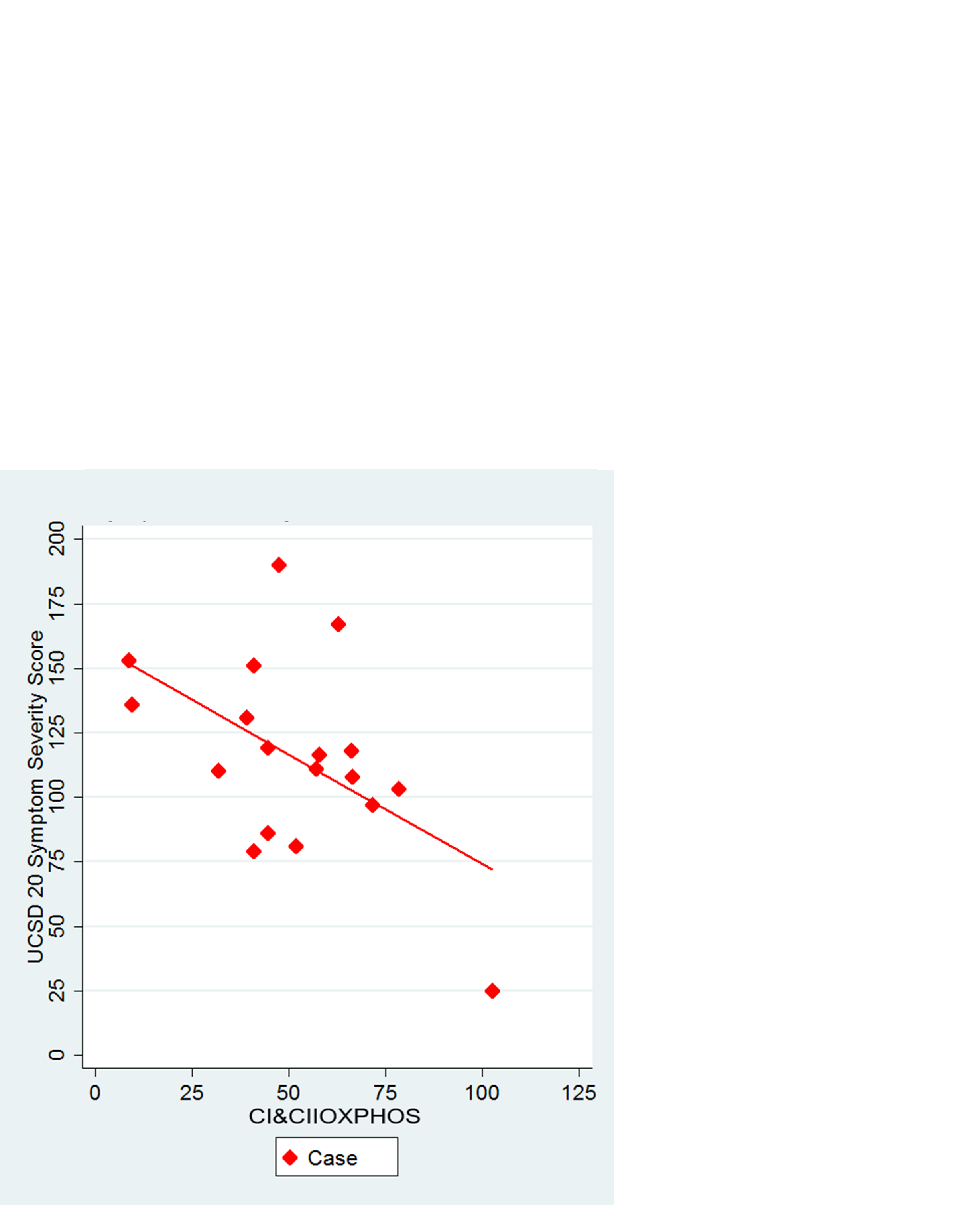
**

Gulf War illness cases separately: Greater symptom severity (y-axis) is tied to lower CI&CIIOXPHOS (mitochondrial function marker).
